# Case report of a 45-year old female Fabry disease patient carrying two alpha-galactosidase A gene mutation alleles

**DOI:** 10.1186/s12881-016-0309-z

**Published:** 2016-07-19

**Authors:** Daniel Oder, Dorothee Vergho, Georg Ertl, Christoph Wanner, Peter Nordbeck

**Affiliations:** Department of Internal Medicine I and Comprehensive Heart Failure Center (CHFC), University Hospital Würzburg, Oberdürrbacher Str. 6, D-97080 Würzburg, Germany; Fabry Center for Interdisciplinary Therapy (FAZIT), University Hospital Würzburg, Würzburg, Germany

**Keywords:** Fabry disease, Cryptogenic stroke, Pain, Hypertrophic cardiomyopathy, Chronic kidney disease

## Abstract

**Background:**

X-chromosomal inheritance patterns and generally rare occurrence of Fabry disease (FD) account for mono-mutational hemizygous male and heterozygous female patients. Female mutation carriers are usually clinically much less severely affected, which has been explained by a suggested mosaicism in cell phenotype due to random allele shutdown. However, clinical evidence is scarce and potential additional effects in female gene carriers, which might account for specific clinical characteristics such as less severe chronic kidney disease, are yet unknown.

**Case presentation:**

This article reports on a 45 year old female patient carrying the two alpha-galactosidase A gene mutations c.416A > G, p.N139S in exon 3 and c.708G > C, p.W236C in exon 5, but still showing only mild organ manifestations.

**Conclusion:**

This current case highlights the importance of careful clinical characterization in patients with Fabry disease, who may show additional rare constellations and, therefore, are in need of personalized medicine. The impact of potential additional protective effects exceeding the presence of a non-pathogenic *GLA* allele in female gene carriers requires further investigation.

## Background

Anderson-Fabry disease (FD) is a rare, X-chromosomal inherited lysosomal storage disorder resulting from currently over 800 known pathogenic alpha-galactosidase A gene (*GLA*) mutations [1, 2]. Recent focus has been set on understanding mutation-specific clinical characteristics and outcome, which might eventually lead to a clinically relevant sub-classification of FD, such as into classical, non-classical, “late onset” and/or organ-specific variants [3–6]. While most X-linked diseases only cause phenotypical manifestations in male patients with females usually being completely unaffected carriers [7, 8], FD-females may develop manifestations to a specific extent, which are usually much less severe in terms of clinical symptoms compared to respective men [2, 9, 10]. This is attributed to the fact that women nearly always present a heterozygous *GLA* genotype including one further, non-pathologically affected allele. However, the definite underlying mechanisms ultimately leading to somewhat less morbidity in female FD patients are still under debate. A potential impact of skewed X-inactivation has been supposed central, leading to random transcriptional silencing of one of both X‐chromosomes in every cell, eventually leading to the typical findings of female genetic mosaicism [8, 11, 12]. Due to the marked variance of FD regarding clinical symptoms, there is high interest to characterize the impact of genotypes in order to embrace patients individualized additive therapeutic needs aside from enzyme replacement therapy (ERT) and improve mechanistic knowledge regarding genotype-phenotype pathophysiology.

## Methods

All patients attending the Fabry Center for Interdisciplinary Therapy (FAZIT) Wurzburg, Germany, undergo a standardized comprehensive clinical, laboratory and imaging examination with special focus on Fabry-related impairments and organ involvement. Cardiac imaging modalities include standard two-dimensional echocardiography, as well as speckle tracking analysis, and cardiac magnetic resonance tomography (MRI), both beneficial for indirect quantification of intramural fibrosis as prominently seen in advanced Fabry cardiomyopathy [13–15]. Investigations also include a thorough investigation of the kidneys including biopsy if suitable, central and peripheral nervous system including brain MRI, skin biopsy, and assessment of sweating capacity, and psychic factors, including assessment of quality of life using SF-36.

## Case presentation

In late 2015, a 45-year-old female patient with genetically proven FD approached FAZIT for specialized clinical evaluation and therapy induction. Molecular gene analysis revealed the atypical situation of a heterozygous female patient carrying two different haplotype variants – c.416A > G, p.N139S in exon 3 and c.708G > C, p.W236C in exon 5, one on each X-chromosomal allele – which both have previously been described as potentially pathogenic [16, 17]. Due to the low frequency of pathogenic *GLA* mutations in the population, comparable respective cases are extremely rare. The initial suspicion for FD in this index patient was raised during a routine ophthalmologist checkup leading to the discovery of Fabry-specific depositions in her cornea at young age of six years. Later on, it was revealed that not only her mother, but also her brother are both affected by the same mutational variant (c.708G > C, p.W236C in exon 5), which was in both relatives clinically related to Fabry-associated acral pain, myocardial hypertrophy, and renal dysfunction. In addition, the index patient’s brother suffered from young-aged stroke at age 45 years and now receives hemodialysis due to end-stage chronic kidney disease. Her grandfather from maternal site is anticipated to have been affected by FD, suffering from fatal end-stage kidney disease at his early forties. Unfortunately, the index patient’s 74 years old father refused to undergo genetic analysis. As the index patient never subjectively suffered from any health problems, neither doctors were consulted nor medication taken until the event of young-aged cryptogenic stroke at the age of 44 years. As a result of stroke, she attended FAZIT for clinical examination and initiation of life-time ERT.

**Fig. 1:**
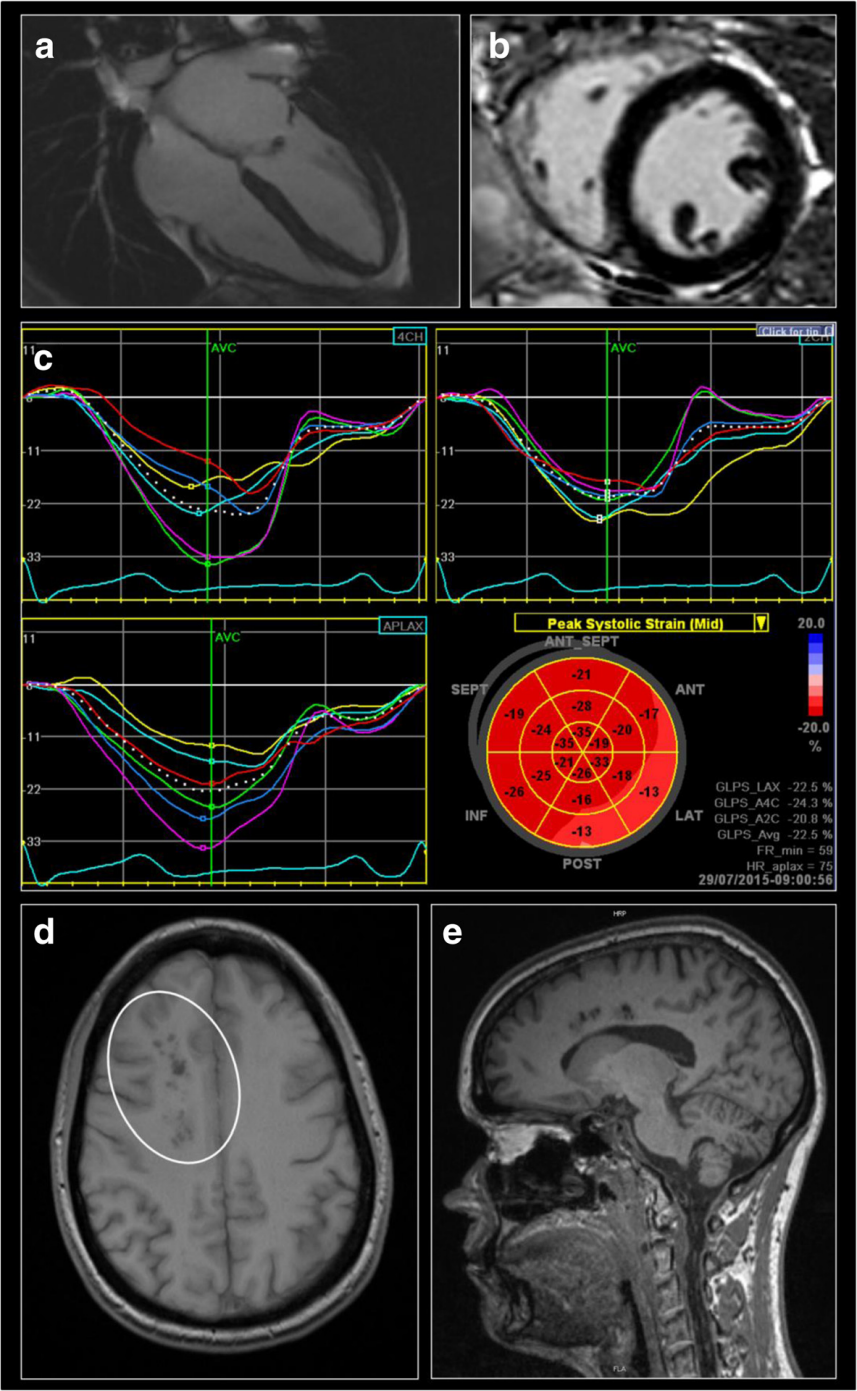
Index patient’s imaging features of cardiac **a**-**c** and brain **d**-**e** involvement at FAZIT baseline visit. Please note that morphologic and late gadolinium enhancement cardiac MRI **a**-**b** reveals mild hypertrophy and marginal fibrotic scar tissue with only minimal intramural late gadolinium enhancement detectable. In two-dimensional speckle tracking **c** peak systolic strain is mildly reduced, visualizing in loco typico early stage Fabry cardiomyopathy in the posterior-lateral and antero-lateral walls (bull’s eye method). Brain MRI **d**-**e** shows residual lesions (encircled) due to previously suffered cryptogenic stroke

At FAZIT she denied any acral pain or gastro-intestinal claims besides of frequent diarrhea. Sweating capacity was reported mildly reduced, but only attracted her attention in hot summer months (Table 1). Alpha-galactosidase A (*α-Gal A*) enzyme activity was measured reduced (0.26 nmol/min/mg protein in leucocytes; reference: 0.4–1.0) and plasma lyso-Gb3 was elevated (30.2 ng/ml; reference: ≤0.9). The body-mass-index (20.5 kg/m^2^), resting blood pressure (100/82 mmHg) and heart rate (80 bpm) were all in normal ranges, with sinus rhythm and no signs of cardiac hypertrophy or ischemia in neither resting, nor exercise electrocardiograms (ECG). (Table 2) Physical capacity in exercise ECG was sufficient, reaching a maximal heart rate of 133 bpm, 75 % of the age-predicted optimum of 176 bpm at 125 W. No spontaneous cardiac arrhythmia was detectable in Holter monitoring, or evocable in exercise stress test. The cardiac biomarker high-sensitive troponin T was inconspicuous for cardiac involvement (<5 pg/ml; reference: 0–14). N-terminal pro-brain natriuretic peptide was slightly elevated (325 pg/ml; reference: <125), which might be a hint on an early stage cardiac involvement. This suspicion from blood biomarkers was supported by findings in standard cardiac imaging, revealing borderline septal and posterior wall thickness of 11 mm and a visually determined concentric left ventricular (LV) hypertrophy with prominent papillary muscles but physiologically preserved LV ejection fraction (68 %) in echocardiography. The normalized LV mass was 66.7 g/m^2^, normalized end-systolic volume 26.0 ml/m^2^, normalized end-diastolic volume 75.9 ml/m^2^, normalized stroke volume 49.9 ml/m^2^, and the cardiac index 3.19 l/min/m^2^ in cardiac MRI with minimal intramural left ventricular late gadolinium enhancement, as seen in early stage Fabry cardiomyopathy (Fig. 1a/b) [15, 18]. In order to more evidently investigate the possible presence of mild fibrotic scar tissue, two-dimensional echo speckle tracking was performed, allowing inferences on myocardial muscle rigidity and stiffness in an 18 segment model. The result revealed a mild pathologic peak systolic strain in the posterior-lateral and anterior-lateral wall segments visualized by speckle tracking bull’s eye (Fig. 1c) hinting on a very early stage of cardiomyopathy [14]. Renal function was completely preserved, with a 99-Technetium DTPA clearance of 90 ml/min, a serum-creatinine of 0.80 mg/dl (reference: 0–0.95), and cystatin-c of 0.76 mg/l (reference: 0.61–0.95), with no prove of pathologic proteinuria in spot or collecting urine. Brain MRI revealed residual lesions due to the previously suffered stroke (Fig. 1d/e), Health related quality of life was accessed by the SF-36 questionnaires and revealed reduced physical (41.61 out of 100) and mental component summary scores (35.17 out of 100) reflecting both somatic and mental impact on the index patient’s subjective well-being.

**Table 1:** General- and Fabry-associated characteristics, biomarkers, renal function and quality of life of the index patient

Variables	Index patient	*Reference values*
Demographics		
Age at first visit (years)	44	
Body-mass-index (kg/m2)	20.7	
Biomarkers		
Lyso-Gb3 (ng/ml)	30.2	<0.9
α-Gal A (nmol/min/mg protein)	0.26	0.4–1.0
NT-proBNP (pg/ml)	325	0–153 (age-dependent)
hs-TnT (pg/ml)	<5	0–14
Renal		
Serum-Creatinine (mg/dl)	0.80	0–0.95
Cystatin C (mg/l)	0.76	0.61–0.95
GFR DTPA Clearance (ml/min)	90	90–150
Fabry associated		
Angiokeratoma	none	
Cornea verticillata	yes	
Impaired sweating	yes	
Vertigo	yes	
Tinnitus	yes	
Frequent diarrhea	yes	
Constipation	none	
Abdominal pain	none	
Abdominal cramps	none	
Nausea/vomiting	none	
Medication		
ERT	Agalsidase beta	
Beta blocker	none	
ACEi/ARBs	none	
Ca-blockers	none	
Diuretics	none	
ASA/OAC	yes	
Quality of life (SF-36 questioner)		
Physical functioning	75	
Role physical	0	
Bodily pain	100	
General health	45	
Vitality	20	
Social functioning	12.5	
Role emotional	100	
Mental health	48	
Physical component summary score	41.61	
Mental component summary score	35.17	

Abbreviations: *α-Gal A*alpha-galactosidase A enzyme activity, *ACEi* angiotensin-converting-enzyme inhibitor, *ARBs* Angiotensin II receptor antagonists, *ASA/OAC* acetylsalicylic acid/oral anticoagulation therapy, *ERT* enzyme replacement therapy, *GFR* glomerular filtration rate, *hs-TnT* high-sensitive troponin T, *NT-proBNP* N-terminal pro-brain natriuretic peptide

**Table 2:** Cardiac features and imaging modalities results in the index patient

Variables	Index patient
Cardio-vascular	
Systolic blood pressure (mmHg)	115
Diastolic blood pressure (mmHg)	75
Heart rate (bpm)	68
Cardiac stress test	
maximal heart rate (bpm)	133
maximal watts (watts)	75
Echocardiography	
LVEF (%)	68
IVSd (mm)	11
LVPWd (mm)	11
LVMI (g/m^2^)	88
E/A	1.3
E/E’	10
DT (ms)	155
Speckle tracking strain [%]	
global strain	−23.44
basal strain	−15.79
mid strain	−21.18
apical strain	−33.74
septal strain	−25.71
lateral strain	−21.43
Speckle tracking strain rate [S^−1^]	
global strain rate	−1.50
basal strain rate	−1.24
mid strain rate	−1.25
apical strain rate	−2.17
septal strain rate	−1.61
lateral strain rate	−1.49
Cardiac MRI	
LGE (yes/no)	none
normalized LVM (g/m^2^)	66.7
normalized ESV (ml/m^2^)	26.0
normalized EDV (ml/m^2^)	75.9
normalized SV (ml/m^2^)	49.9
CI (l/min/m^2^)	3.19
LVEF (%)	65.7

*Abbreviations*: *DT* deceleration time, *IVSd* interventricular septum thickness in end-diastole, *LVEF* left ventricular ejection fraction in %, *LVMI* left ventricular mass index in echocardiography, *LVPWD* left ventricular posterior wall thickness in end-diastole, *normalized EDV* normalized end-diastolic volume in cardiac MRI, *normalized ESV* normalized end-systolic volume in cardiac MRI, *normalized LVM* normalized left ventricular mass in cardiac MRI, *normalized SV* normalized stroke volume in cardiac MRI

By rules of genetic penetrance, in this particular case the index patient conducts one of her pathogenic *GLA* alleles to all of her biological descendants. Thus both of her children, an 11 year old daughter and her 8 year old son underwent genetic analysis for FD (Centogene AG, Rostock, Germany), both presenting the c.708G > C, p.W236C mutation in exon 5. *α-Gal A* enzyme activity was reduced in her daughter (0.20 nmol/min/mg protein in leucocytes) and highly reduced in the index patient’s son (0.03 nmol/min/mg protein in leucocytes). Lyso-Gb3 was 8.1 ng/ml in the index patient’s daughter and not determined in her son. Interestingly, her son already claims about stinging pain occurring in situations of bodily stress and during infections even though he is at very young age.

Female mutation carriers usually present milder phenotypes than comparable males, which might be explained due to compensatory effects of the second, non-pathologically affected allele. In rare cases of homozygous female patients classical FD is to be expected. In this regard, Rodríguez-Marí and colleagues reported about a young female patient, who was found homozygous for the Q279R *GLA* mutation and presented a classics Fabry-phenotype with cardiac and neurological organ involvement, reduced *α-Gal A* activity, Fabry-associated angiokeratoma, a reduced sweating capacity and acral pain, which started at young age of 8 years [19]. In contrast, all three female homozygous patients (p.Arg118Cys variant) published by Susana Ferreira and colleagues did not develop a classical Fabry phenotype, highlighting the impact of mutation-specific factors in FD [20]. However, contradictory to both mentioned reports, the index patient of the current study was found homozygous with not one and the same, but two different *GLA* mutations in each of her alleles. Regarding our index patient, family pedigree, laboratory data, and clinical manifestations give a mixed picture regarding disease patterns. While young-aged stroke gives evidence for neurological manifestations, further organ involvement was minimal with only very mild cardiac and no renal impairments detectable. Comparing her clinical course to her brothers’, much less severe manifestations were found. This could indicate that there might not be an additive effect of two independent pathogenic *GLA* alleles, questioning the anticipated clinical impact of skewed X-inactivation leading to silence of the index patient’s second pathogenic allele. It might instead be speculated that there could be so far unknown additional modifying effects in females, preventing severe clinical courses including e.g. chronic renal disease. These assumptions remain limited due to the scarce comprehensive data about p.N139S available in literature, discussing this respectively novel mutation being of pathogenic impact [17]. However, long-term results remain to be evaluated in order to judge on clinical severity and outcome over time.

Both of the index patient’s children are likely to develop organ involvement as seen in their biological relatives. As therapeutic effects of ERT have been reported to be most beneficial when started before organ injury is detectable [21, 22], it is to be discussed whether and when the index patient’s children should start receiving ERT, particularly in the light of the high prevalence of young-aged stroke in the family pedigree.

## Conclusion

In summary, this current case highlights the importance of careful clinical characterization in patients with Fabry disease, who may show additional uncommon constellations and are thus in need of personalized medicine. The impact of potential additional protective effects exceeding the presence of a non-pathogenic *GLA* allele in female gene carriers require further investigation.

## Abbreviations

*α-Gal A,* alpha-galactosidase A; ERT, enzyme replacement therapy; FAZIT, Fabry Center for Interdisciplinary Therapy Würzburg; FD, Fabry disease; *GLA,* alpha-galactosidase A encoding gene; LV, left ventricle of the heart.
